# Interactome dynamics of RAF1-BRAF kinase monomers and dimers

**DOI:** 10.1038/s41597-023-02115-0

**Published:** 2023-04-12

**Authors:** Luis F. Iglesias-Martinez, Nora Rauch, Kieran Wynne, Brendan McCann, Walter Kolch, Jens Rauch

**Affiliations:** 1 Systems Biology Ireland, School of Medicine, University College Dublin, Dublin, Ireland; 2 Conway Institute of Biomolecular and Biomedical Research, University College Dublin, Dublin, Ireland; 3 School of Biomolecular and Biomedical Science, University College Dublin, Dublin, Ireland; 4 Present Address: Voscuris Ltd., Jefferson House 42 Queen Street, Belfast, BT1 6HL United Kingdom

**Keywords:** Protein-protein interaction networks, Oncogenes

## Abstract

RAF kinases play major roles in cancer. BRAFV600E mutants drive ~6% of human cancers. Potent kinase inhibitors exist but show variable effects in different cancer types, sometimes even inducing paradoxical RAF kinase activation. Both paradoxical activation and drug resistance are frequently due to enhanced dimerization between RAF1 and BRAF, which maintains or restores the activity of the downstream MEK-ERK pathway. Here, using quantitative proteomics we mapped the interactomes of RAF1 monomers, RAF1-BRAF and RAF1-BRAFV600E dimers identifying and quantifying >1,000 proteins. In addition, we examined the effects of vemurafenib and sorafenib, two different types of clinically used RAF inhibitors. Using regression analysis to compare different conditions we found a large overlapping core interactome but also distinct condition specific differences. Given that RAF proteins have kinase independent functions such dynamic interactome changes could contribute to their functional diversification. Analysing this dataset may provide a deeper understanding of RAF signalling and mechanisms of resistance to RAF inhibitors.

## Background & Summary

RAF kinases are critical downstream effectors of oncogenic RAS. They bind to and phosphorylate the dual specificity kinases MEK1/2, which in turn activate ERK1/2 kinases. While ERK has hundreds of substrates^1^, the RAF-MEK-ERK cascade is perceived as a linear amplifier with negative feedback that adjusts the signalling amplitude and kinetics of ERK^2^. BRAF is mutated in ~6% of all human cancer and with very high frequency in hairy cell leukaemia (>90%), papillary thyroid cancer (~70%) and malignant melanomas (40–50%)^3^. The most common mutation is V600E in the activation loop. Several potent RAF kinase inhibitors targeting BRAFV600E are in clinical use but suffer from a high frequency from resistance development^4^. The mechanisms of RAF inhibitor resistance are well studied, and in most case rely on the reactivation of ERK signalling^4^. One of the most common resistance mechanisms is BRAF heterodimerization with RAF1^5^. RAF dimers can maintain strong MEK and ERK activation known as paradoxical activation^6–9^. A drug bound RAF protomer can allosterically activate the other protomer, while thermodynamic and structural constraints prevent the RAF inhibitor to also bind to and inhibit the activated protomer^10,11^.

Thus, RAF dimerization has attracted considerable interest. Here, we investigated whether RAF dimers only enhance and maintain ERK pathway signalling or whether they can also diversify signalling to other pathways. This question has not been addressed, as all attention was focussed on re-activation of ERK signalling. However, RAF1 - and probably also BRAF – have important kinase independent functions that are exerted through protein-protein interactions. For instance, RAF1 can block apoptosis by binding to and inhibiting the function of ASK1^12^, MST2^13^, and ROKα^14^ in a kinase independent manner. The pathophysiological relevance of these interactions was recently demonstrated in a mouse model of lung cancer, which showed that RAF1 but not its catalytic activity is required for tumour progression^15^. Therefore, changes in protein-protein interactions could be important for the function of RAF monomers and dimers. In order to investigate this, we mapped the interactomes of the RAF1 monomer, and the dimers of RAF1 with BRAF or BRAFV600E using quantitative mass spectrometry. In addition, we measured the effects of vemurafenib and sorafenib, two clinically used RAF inhibitors that bind to different inactive conformations of BRAF: vemurafenib to the DGF in and αC helix out position, and sorafenib to the DGF out and αC helix in position^4^. Stabilizing different protein conformations could plausibly change the interactome and contribute to drug effects independent of catalytic inhibition. This aspect is usually neglected, but could potentially explain some of the inhibitor effects, and more importantly lend itself to improve the design of kinase inhibitors by including considerations of interactome changes.

**Fig. 1:**
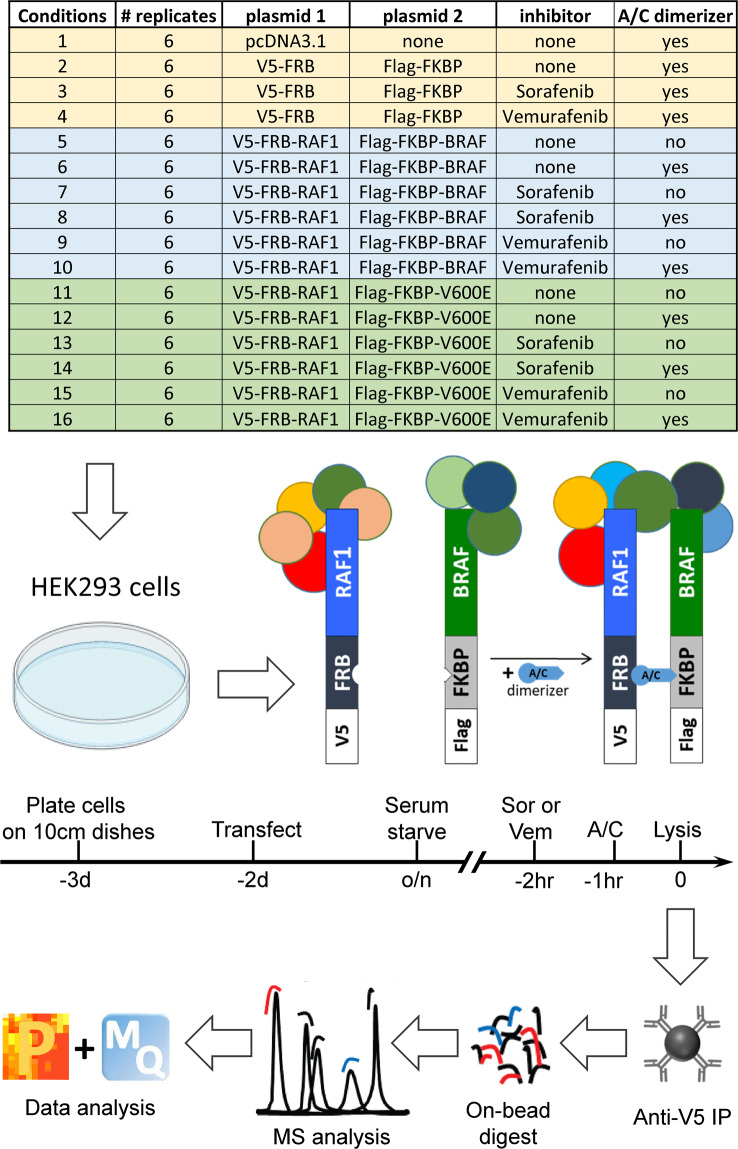
Overview of the experimental workflow. See text for details. Abbreviations: Sor, Sorafenib; Vem, Vemurafenib; A/C, A/C heterodimerizer drug; MQ, Maxquant; P, Perseus.

BRAF and RAF1 kinases homo- and heterodimerize as part of the normal activation cycle^16,17^. Most RAF kinase inhibitors induce and enhance RAF dimerization albeit to different extents^4^. As RAF kinases can naturally dimerize^18^ co-transfection of RAF1 with BRAF generates a small amount of heterodimers that increased fourfold when cells are treated with dimerizer (Fig. 3A). BRAFV600E has a stronger tendency to heterodimerize with RAF1 than wildtype BRAF^18^ resulting in a twofold increase of heterodimers and a sevenfold induction by the dimerizer drug. Thus, while there is a contribution of BRAF proteins to the RAF1 interactome in uninduced (no dimerizer drug) conditions, it is only 25–27%. In order to avoid confounding issues arising from different stoichiometries and instability of RAF dimers during immunoprecipitation we used iDimerize, an artificial system for directed protein dimerization^19^. iDimerize uses an asymmetric drug (AP21967, also termed A/C) that specifically crosslinks FRB and FKBP protein domains with high affinity and very long dissociation times that allows the isolation of stable protein dimers by immunoprecipitation. The FRB and FKBP domains used are mutated so that the A/C drug specifically crosslink the artificial FRB/FKBP domains but not endogenous proteins containing such domains. This regime allowed us to systematically map the interactome of RAF1 monomers, RAF1-BRAF, and RAF1-BRAFV600E dimers. The workflow is depicted in Fig. 1.

**Fig. 2:**
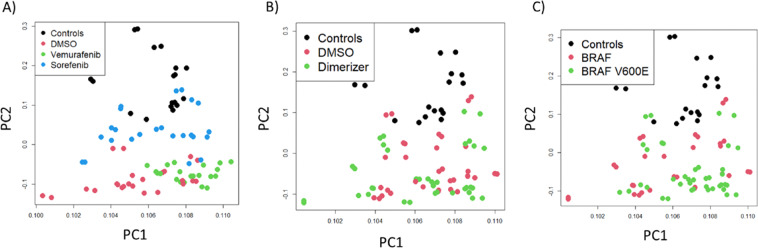
Principal Component Analysis (PCA) showing the effects of each condition on sample separation. (**A**) Separation by Vemurafenib and Sorafenib treatments. Control, transfection with V5-FRB and Flag-FKBP; DMSO, vehicle control. (**B**) Separation by dimerization. A/C, samples treated with A/C dimerizer drug; samples treated with DMSO. (**C**) Effects of expression of BRAF vs. BRAFV600E.

There was a large overlap between conditions, but also differential binding of proteins between monomer and dimer states supporting our hypothesis that differential protein-protein interactions (PPIs) may contribute to signalling diversification. (Table 1). The number of RAF interacting proteins was generally decreased with Sorafenib, while Vemurafenib had a lesser impact. Interestingly, principal component analysis (PCA) showed that Sorafenib and Vemurafenib treatments separated samples best (Fig. 2) suggesting that RAF inhibitors may have a major impact on non-catalytic RAF signalling.

**Table 1:** Overlap of interacting proteins between conditions.

RAF1 +	BRAF	BRAF + A/C	BRAF + S	BRAF + S + A/C	BRAF + V	BRAF + V + A/C	V600E	V600E + A/C	V600E + S	V600E + S + A/C	V600E + V	V600E + V + A/C
BRAF	75/75	55/75	17/75	19/75	26/75	35/75	30/75	49/75	20/75	15/75	21/75	26/75
BRAF + A/C	55/118	118/118	17/118	25/118	29/118	47/118	29/118	65/118	22/118	14/118	20/118	30/118
BRAF + S	17/25	17/25	25/25	20/25	20/25	21/25	16/25	21/25	21/25	17/25	14/25	19/25
BRAF + S + A/C	19/41	25/41	20/41	41/41	20/41	27/41	15/41	25/41	24/41	18/41	14/41	21/41
BRAF + V	26/51	29/51	20/51	20/51	51/51	45/51	22/51	29/51	19/51	15/51	26/51	37/51
BRAF + V + A/C	35/88	47/88	21/88	27/88	45/88	88/88	23/88	43/88	26/88	18/88	39/88	47/88
V600E	30/31	29/31	16/31	15/31	22/31	23/31	31/31	30/31	16/31	15/31	17/31	22/31
V600E + A/C	49/78	65/78	21/78	25/78	29/78	43/78	30/78	78/78	22/78	16/78	17/78	28/78
V600E + S	20/33	22/33	21/33	24/33	19/33	26/33	16/33	22/33	33/33	18/33	15/33	20/33
V600E + S + A/C	15/21	14/21	17/21	18/21	15/21	18/21	15/21	16/21	18/21	21/21	13/21	16/21
V600E + V	21/47	20/47	14/47	14/47	26/47	39/47	17/47	17/47	15/47	13/47	47/47	26/47
V600E + V + A/C	26/54	30/54	19/54	21/54	37/54	47/54	22/54	28/54	20/54	16/54	26/54	54/54

A/C, dimerizer drug; S, Sorafenib; V, Vemurafenib. The denominator represents the number of interactors in the row, while the numerator is the number of interactors also found in the condition described in the column.

## Methods

### Construction of iDimerize plasmids

Plasmids for the induced dimerization of RAF1 and BRAF were generated in two steps.

Using the iDimerize plasmids pC_4_-RHE and pC_4_EN-F1 (former ARIAD Pharmaceuticals, now Takara Bio)^19^ as template, the ORFs encoding FRB and FKBP respectively were amplified by PCR and cloned into pcDNA3.1(+) (Invitrogen), giving the pFRB and pFKBP vectors. For detection and immunoprecipitation of the fusion proteins, N-terminal tags were added to FRB (V5) and FKBP (FLAG). Subsequently, the ORFs of RAF1 and BRAF (wildtype/V600E) were cloned into pFRB and pFKBP, respectively, C-terminally of the dimerization domain.

### Cells and transfection

HEK293 cells were grown in Dulbecco’s modified eagle medium (DMEM, Gibco) supplemented with 10% (v/v) foetal bovine serum and 2mM L-glutamine at 37 °C in a humidified atmosphere containing 5% CO_2_. Cells were transfected one day after seeding at 50–70% confluence using Lipofectamine 2000 reagent (Invitrogen) according to the manufacturer’s instructions. Cells were serum starved overnight before treatment with dimerizer and drugs as indicated in Fig. 1.

### Cell lysis, immunoprecipitation and Western blotting

Cells were lysed in ice cold 10 mM Tris pH 7.5, 150 mM NaCl, 0.5% NP40 supplemented with protease and phosphatase inhibitors (Roche Diagnostics, 1 tablet/10 ml). The lysates were cleared of debris by centrifugation at 15,000 × g for 10 min at 4 °C. The protein concentrations in supernatants were measured using the Pierce® BCA Protein Assay Kit according to the manufacturer’s instructions. Lysates were adjusted to contain equal amounts of protein and used for immunoprecipitation and Western blot analysis. Antibodies used were anti-Flag (Clone M2, F3165 from Sigma-Aldrich), anti-V5-HRP (46-0705 from Invitrogen), anti-ERK1/2 (M5670 from Sigma-Aldrich), anti-phospho-ERK (M8159 from Sigma-Aldrich). Sorafenib tosylate (Selleckchem, #S1040) and Vemurafenib (PLX4032, Selleckchem, #S1267) were used at 30 µM final concentration. A/C Heterodimerizer AP21967 (previously Agilent, now Takara, #635056) was used at 0.5 µM.

### Affinity purification – mass spectrometry (AP-MS)

All samples were prepared as 3 biological replicates, each with 2 technical replicates. Cleared lysates were immunoprecipitated with 5μl V5 agarose beads for 30 minutes at 4 °C. Beads were washed 3 times with 500 μl ice-cold IP wash (10 mM Tris pH7.5, 150 mM NaCl). Then, tryptic peptides were obtained by on bead digestion^20^. Briefly, the immunoprecipitates were incubated in 60 μl of Buffer 1 (2 M urea, 50 mM Tris-HCl pH 7.5, 5 μg/ml Trypsin [modified sequencing-grade trypsin; Promega]) for 30 minutes at 37 °C. Beads were washed twice in 25 μl of buffer 2 (2 M urea, 50 mM Tris-HCl pH 7.5, 1 mM DTT). The supernatants were pooled and left to digest overnight at room temperature. Then, samples were alkylated by addition of 20 μl iodoacetamide (5 mg/ml) and incubation for 30 minutes in the dark. The reaction was stopped by adding 1 μl 100% trifluoroacetic acid (TFA), and samples were desalted using C_18_ StageTips containing octadecyl C_18_ disks (Supelco, Sigma, United Kingdom) as previously described^21^. The eluates were concentrated in a CentriVap concentrator (Labconco, USA) and re-suspended in 12 μl 0.1% TFA. MS-analysis was performed as previously reported^20^. Tryptic peptides were separated on an Ultimate Ultra3000 chromatography system using homemade columns (100 mm length, 75 mm inside diameter packed with 1.8 μm RepreosilAQ C_18_ (Dr Maisch, Germany)) and a 40 minute 3%–32% acetonitrile gradient at a flow rate of 200 nl/minute. Peptides were identified and quantified using a Thermo Scientific Q Exactive operated in positive ion mode with a potential of 2,000 V applied to the column and a capillary temperature of 220 °C. The mass spectrometer operated in automatic data-dependent switching mode, selecting the 12 most intense ions prior to tandem MS (MS/MS) analysis.

### MS data analysis

MaxQuant (Version 1.3.0.5) was used to analyse raw mass spectrometric data files from LC-MS/MS for label free quantification (LFQ). Default settings were used unless stated otherwise, including the following parameters: Trypsin/P digest; variable modifications included Oxidation (Met) and Acetyl (Protein N-term); fixed modification included Carbamidomethyl (Cys) only; multiplicity = 1; first search at 20 ppm; main search at 6 ppm mass accuracy (MS) and 20 mass deviation for the fragment ions; data searched against a human database (Uniprot HUMAN); minimum peptide length of 6; unfiltered for labelled amino acids; false discovery rate (FDR) of 0.01 selected for peptides and proteins; results refined through re-quantify option; match between runs selected with 1 min time window; label free quantification (LFQ) with LFQ minimum ratio count set at 1. LFQ values for proteins identified by MaxQuant were further analysed using the Perseus software^22,23^. The following categories were retained from the MaxQuant proteinGroups.txt file: LFQ intensity for each replicate; Unique Peptides; Sequence coverage [%]; Identified only by site; Reverse; Contaminant; Mol. Weight [kDa]; Protein IDs; Majority protein IDs; Protein names; Gene names; Proteins. The proteinGroups list was filtered to remove proteins identified in the following categories: Only Identified by site (peptides identified by a modification site only); Reverse (identified in reversed part of decoy database); Contaminant (known contaminants). The LFQ intensities for each replicate were log base 2 transformed. The log of an LFQ intensity of zero is not a valid value and is replaced by NaN (not a number). A categorical row annotation was then created to group replicates into their respective time points (Control, 0 min, 5 min, 15 min, 30 min, 120 min). The data was then filtered to keep only those rows that contained at least 3 valid values in at least 1 group (time point). NaN was then replaced with valid values, thus enabling statistical analysis. Missing values were imputed based on the following parameters: Width – 0.3; Down Shift – 1.8; Mode – Separately for each column. Right-sided t-tests were performed to compare each time point group with the control using permutation-based FDR for truncation. This produced a –log p value statistic and a t-test difference value. These were visualised as volcano plot with the x-axis representing t-test difference and the y-axis –log p value. Perseus allows an adjustable cut off curve to be plotted that allows the importance given to the –log p value and t-test difference to be adjusted. Based on visual interpretation of the data, the position of the cut off curve is adjusted by varying the False Discovery Rate (FDR) and Artificial Within Group Variance (s0). FDR of 0.1 and s0 of 0.1 were selected and used as the cut off curve for each time point and set at 250 randomisations. All points to the right (positive t-test difference) were deemed significant.

### Normalization and missing values imputation

Proteins with more than 2 missing values in each condition were discarded. Proteins with less than 2 missing values per condition were retained and the missing values were imputed using random samples from a log normal distribution. The parameters of the log normal distribution were inferred from the non-missing values in the replicates of the same condition. After the imputation the data was log transformed. Two samples were filtered out after a PCA indicated they were outliers. The outliers were one biological replicate transfected with BRAFV600E and treated with Sorafenib and the dimerizer.

### Differentially expressed protein analysis

We used a one tailed t-test to see which proteins were more abundant in a treatment case against their control. The resulting P-values were FDR adjusted. We deemed as statistically significant proteins with a fold change higher than 1.5 and FDR adjusted P-value lower than 0.05.

### Linear regression analysis

We used a linear model to assess the contribution of each variable to the LFQ intensities. We used a one-hot encoding for five variables: one variable to represent whether a sample was transfected with wild-type BRAF, the second for BRAF V600E mutant transfection, the third if it was treated with dimerizer, and the fourth and fifth for treatment with sorafenib and vemurafenib, respectively. The coefficients in the linear regression indicate how each of these variables affect log-LFQ values. If a variable’s coefficient is positive, then that variable increases the log-LFQ value and vice versa. We used a t-test on the coefficients to determine if they were statistically significant from zero. The P-values were FDR adjusted. Finally, we used the limma package in R to do a contrast analysis, i.e. calculating the difference in regression coefficients between variables. A positive contrast sign indicates that a variable is increasing the log-LFQ value of an interacting protein under this condition, and vice versa.

## Data Records

All interactors identified and quantified by mass spectrometry proteomics data were submitted to the ProteomeXchange Consortium via the PRIDE^24^ partner repository with the dataset identifier PXD036792^25^ and to the IMEx (http://www.imexconsortium.org) consortium through IntAct^26^ with the identifier IM-29658^27^. The deposited data comprise both raw files and Maxquant processed files and are described in Table 2. The submitted data also contain AP-MS experiments with KSR1, a scaffold protein and dimerization partner for RAF1 and BRAF^28^, and with BRAF D594G, which is a low-activity oncogenic BRAF mutant^29^. These data are not discussed in the text as we did not perform validation experiments for these datasets. Differential interactors identified and quantified by log fold change are reported in the Table “Differential interactors identified and quantified by log fold change” deposited at Figshare^30^. The results of the linear regression and contrast analysis are shown in the Table “Contrast analysis” also deposited at Figshare^30^. This table compares proteins differentially interacting between RAF1 monomers and dimers formed with BRAF and BRAF upon A/C dimerizer treatment; proteins differentially interacting between BRAF and BRAFV600E transfected samples; and the influence of sorafenib and vemurafenib treatments on the RAF interactome.

**Table 2:** Guide to data files deposited in PRIDE Database.

Raw Data Files:						
Construct(s)		Drug	A/C	Biological Replicate 1	Biological Replicate 2	Biological Replicate 3
**a) Data used in the publication**						
pcDNA3.1		−	+	29AprCR_Nora1.raw	29AprCR_Nora25.raw	29AprCR_Nora49.raw
				29AprCR_Nora1_1_130430110103.raw	29AprCR_Nora25_1.raw	29AprCR_Nora49_1.raw
pFKBP	pFRB	−	+	29AprCR_Nora2.raw	29AprCR_Nora26.raw	29AprCR_Nora50.raw
				29AprCR_Nora2_1_130430142009.raw	29AprCR_Nora26_1.raw	29AprCR_Nora50_1.raw
pFKBP	pFRB	SOR	+	29AprCR_Nora3_1.raw	29AprCR_Nora27.raw	29AprCR_Nora51.raw
				29AprCR_Nora3_130430160530.raw	29AprCR_Nora27_1.raw	29AprCR_Nora51_1.raw
pFKBP	pFRB	VEM	+	29AprCR_Nora4.raw	29AprCR_Nora28.raw	29AprCR_Nora52.raw
				29AprCR_Nora4_1.raw	29AprCR_Nora28_1.raw	29AprCR_Nora52_1.raw
BRAF(wt)	RAF1	−	−	29AprCR_Nora5.raw	29AprCR_Nora29.raw	29AprCR_Nora53.raw
				29AprCR_Nora5_1.raw	29AprCR_Nora29_1.raw	29AprCR_Nora53_1.raw
BRAF(wt)	RAF1	−	+	29AprCR_Nora6.raw	29AprCR_Nora30.raw	29AprCR_Nora54.raw
				29AprCR_Nora6_1.raw	29AprCR_Nora30_1.raw	29AprCR_Nora54_1.raw
BRAF(wt)	RAF1	SOR	−	29AprCR_Nora7.raw	29AprCR_Nora31.raw	29AprCR_Nora55.raw
				29AprCR_Nora7_1.raw	29AprCR_Nora31_1.raw	29AprCR_Nora55_1.raw
BRAF(wt)	RAF1	SOR	+	29AprCR_Nora8.raw	29AprCR_Nora32.raw	29AprCR_Nora56.raw
				29AprCR_Nora8_1.raw	29AprCR_Nora32_1.raw	29AprCR_Nora56_1.raw
BRAF(wt)	RAF1	VEM	−	29AprCR_Nora9.raw	29AprCR_Nora33.raw	29AprCR_Nora57.raw
				29AprCR_Nora9_1.raw	29AprCR_Nora33_1.raw	29AprCR_Nora57_1.raw
BRAF(wt)	RAF1	VEM	+	29AprCR_Nora10.raw	29AprCR_Nora34.raw	29AprCR_Nora58.raw
				29AprCR_Nora10_1.raw	29AprCR_Nora34_1.raw	29AprCR_Nora58_1.raw
BRAF(V600E)	RAF1	−	−	29AprCR_Nora11.raw	29AprCR_Nora35.raw	29AprCR_Nora59.raw
				29AprCR_Nora11_1.raw	29AprCR_Nora35_1.raw	29AprCR_Nora59_1.raw
BRAF(V600E)	RAF1	−	+	29AprCR_Nora12.raw	29AprCR_Nora36.raw	29AprCR_Nora60.raw
				29AprCR_Nora12_1.raw	29AprCR_Nora36_1.raw	29AprCR_Nora60_1.raw
BRAF(V600E)	RAF1	SOR	−	29AprCR_Nora13.raw	29AprCR_Nora37.raw	29AprCR_Nora61.raw
				29AprCR_Nora13_1.raw	29AprCR_Nora37_1.raw	29AprCR_Nora61_1.raw
BRAF(V600E)	RAF1	SOR	+	29AprCR_Nora14.raw	29AprCR_Nora38.raw	29AprCR_Nora62.raw
				29AprCR_Nora14_1.raw	29AprCR_Nora38_1.raw	29AprCR_Nora62_1.raw
BRAF(V600E)	RAF1	VEM	−	29AprCR_Nora15.raw	29AprCR_Nora39.raw	29AprCR_Nora63.raw
				29AprCR_Nora15_1.raw	29AprCR_Nora39_1.raw	29AprCR_Nora63_1.raw
BRAF(V600E)	RAF1	VEM	+	29AprCR_Nora16.raw	29AprCR_Nora40.raw	29AprCR_Nora64.raw
				29AprCR_Nora16_1.raw	29AprCR_Nora40_1.raw	29AprCR_Nora64_1.raw
**b) Data not used in the publication**						
BRAF(D594G)	RAF1	−	−	29AprCR_Nora17.raw	29AprCR_Nora41.raw	29AprCR_Nora65.raw
				29AprCR_Nora17_1.raw	29AprCR_Nora41_1.raw	29AprCR_Nora65_1.raw
BRAF(D594G)	RAF1	−	+	29AprCR_Nora18.raw	29AprCR_Nora42.raw	29AprCR_Nora66.raw
				29AprCR_Nora18_1.raw	29AprCR_Nora42_1.raw	29AprCR_Nora66_1.raw
KSR1	RAF1	−	−	29AprCR_Nora19.raw	29AprCR_Nora43.raw	29AprCR_Nora67.raw
				29AprCR_Nora19_1.raw	29AprCR_Nora43_1.raw	29AprCR_Nora67_1.raw
KSR1	RAF1	−	+	29AprCR_Nora20.raw	29AprCR_Nora44.raw	29AprCR_Nora68.raw
				29AprCR_Nora20_1.raw	29AprCR_Nora44_1.raw	29AprCR_Nora68_1.raw
KSR1	RAF1	SOR	−	29AprCR_Nora21.raw	29AprCR_Nora45.raw	29AprCR_Nora69.raw
				29AprCR_Nora21_1.raw	29AprCR_Nora45_1.raw	29AprCR_Nora69_1.raw
KSR1	RAF1	SOR	+	29AprCR_Nora22.raw	29AprCR_Nora46.raw	29AprCR_Nora70.raw
				29AprCR_Nora22_1.raw	29AprCR_Nora46_1.raw	29AprCR_Nora70_1.raw
KSR1	RAF1	VEM	−	29AprCR_Nora23.raw	29AprCR_Nora47.raw	29AprCR_Nora71.raw
				29AprCR_Nora23_1.raw	29AprCR_Nora47_1.raw	29AprCR_Nora71_1.raw
KSR1	RAF1	VEM	+	29AprCR_Nora24.raw	29AprCR_Nora48.raw	29AprCR_Nora72.raw
				29AprCR_Nora24_1.raw	29AprCR_Nora48_1.raw	29AprCR_Nora72_1.raw
**c) Processed Data: MaxQuant (Version 1.3.0.5) Output Files**						
mqpar.xml						
summary.txt						
experimentalDesignTemplate.txt						
peptides.txt						
checksum.txt						
parameters.txt						
proteingroups.txt						
modificationSpecificPeptides.txt						

(Co-)transfected constructs: pcDNA3.1 (empty vector, Invitrogen), pFKBP (empty vector), B-Raf (wt) – BRAF(wildtype) in pFKBP, B-Raf (V600E) – BRAF(V600E) in pFKBP, B-Raf (D594G) – BRAF(D594G) in pFKBP, KSR1 – KSR1(wildtype) in pKBP, pFRB (empty vector), Raf-1 – RAF1(wildtype) in pFRB. RAF inhibitor drugs: SOR – Sorafenib (30 μM), VEM – Vemurafenib (30 μM), A/C – A/C Heterodimerizer AP21967 (0.5 μM, Agilent). Biological Replicate 1/2/3: Filename (_1 denotes the second technical replicate)

## Technical Validation

**Fig. 3:**
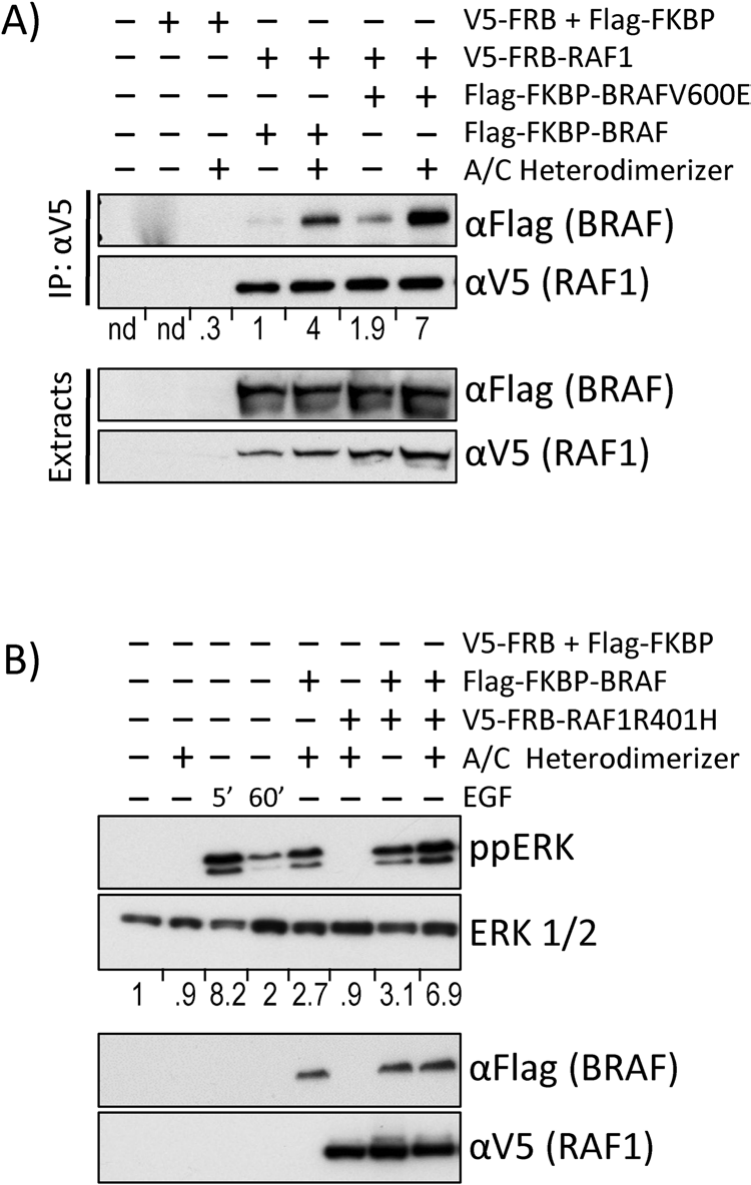
A/C drug treatment induces RAF dimerization and ERK activation. Cells transfected with the indicated plasmids were treated as indicated and proteins were analysed by Western blotting. Western blots were quantified by Image J. (**A**) A/C induces efficient heterodimerization between RAF1 + BRAF and RAF1 + BRAFV600E. IP, immunoprecipitation. Co-precipitating BRAF or BRAFV600E bands were normalized to the respective RAF1 bands. The V5-FRB + Flag-FKBP bands were normalized to background. n.d., not determined. (**B**) RAF heterodimerization induces similar levels of ERK activity (ppERK) as treatment with epidermal growth factor (EGF). Normalised ERK activation (ratio ppERK/ERK) is shown below the ERK blot.

To assure that the A/C heterodimerizer system mimics physiological RAF activation we tested RAF1-BRAF heterodimerization and ERK pathway activation (Fig. 3). Treatment of cells with the A/C heterodimerizer drug efficiently induced RAF1-BRAF and RAF1-BRAFV600E dimerization (Fig. 3A). Transfecting BRAF induced ERK activation, which was further enhanced by treatment with A/C heterodimerizer to levels that are comparable to ERK activation stimulated by treatment with epidermal growth factor (EGF) (Fig. 3B). In order to eliminate potentially confounding effects from physiological dimerization we used a RAF1R401H mutant, which due to a mutation in the interaction interface cannot dimerize^31^. On its own, RAF1R401H was unable to activate ERK. It also could not enhance BRAF induced ERK activation unless cells were treated with A/C heterodimerizer, which induced strong ERK activation similar to EGF. These data suggest that the A/C heterodimerizer system faithfully replicates the physiological RAF activation process.

**Fig. 4:**
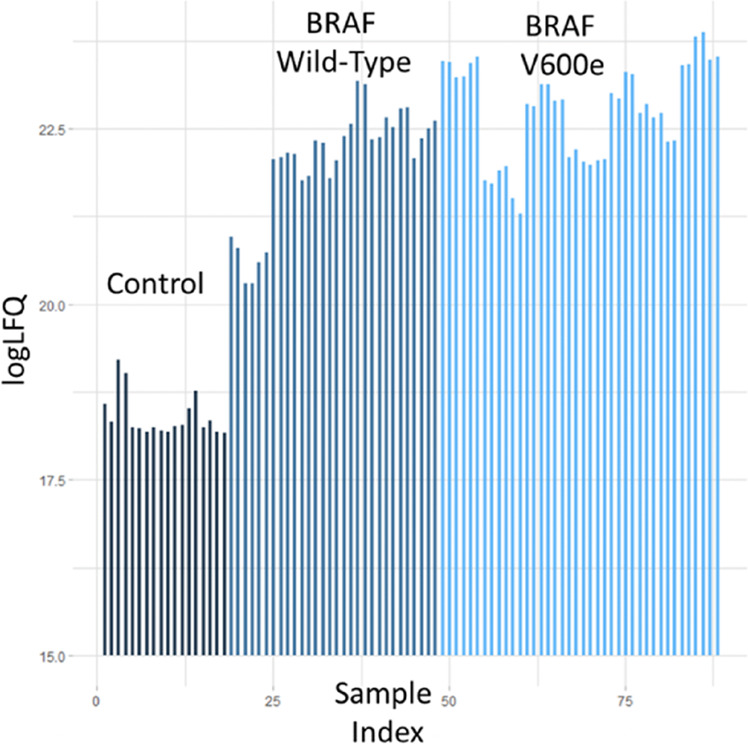
Global estimation of specifically interacting proteins. Samples transfected with V5-FRB-RAF1 + Flag-FKBP-BRAF or V5-FRB-RAF1 + Flag-FKBP-BRAFV600E show higher LFQ intensities across all conditions than samples transfected with V5-FRB + Flag-FKBP controls. Each bar represents a condition.

To assure statistical power three independent biological experiments were performed, and two technical replicates of each experiment were analysed by MS. A comparison of the label free quantitation (LFQ) intensities between control transfections with FRB and FKBP versus RAF1 and BRAF transfections showed a clear enrichment of interacting proteins in the RAF transfected samples (Fig. 4) supporting the validity of the experimental analysis strategy.

**Fig. 5:**
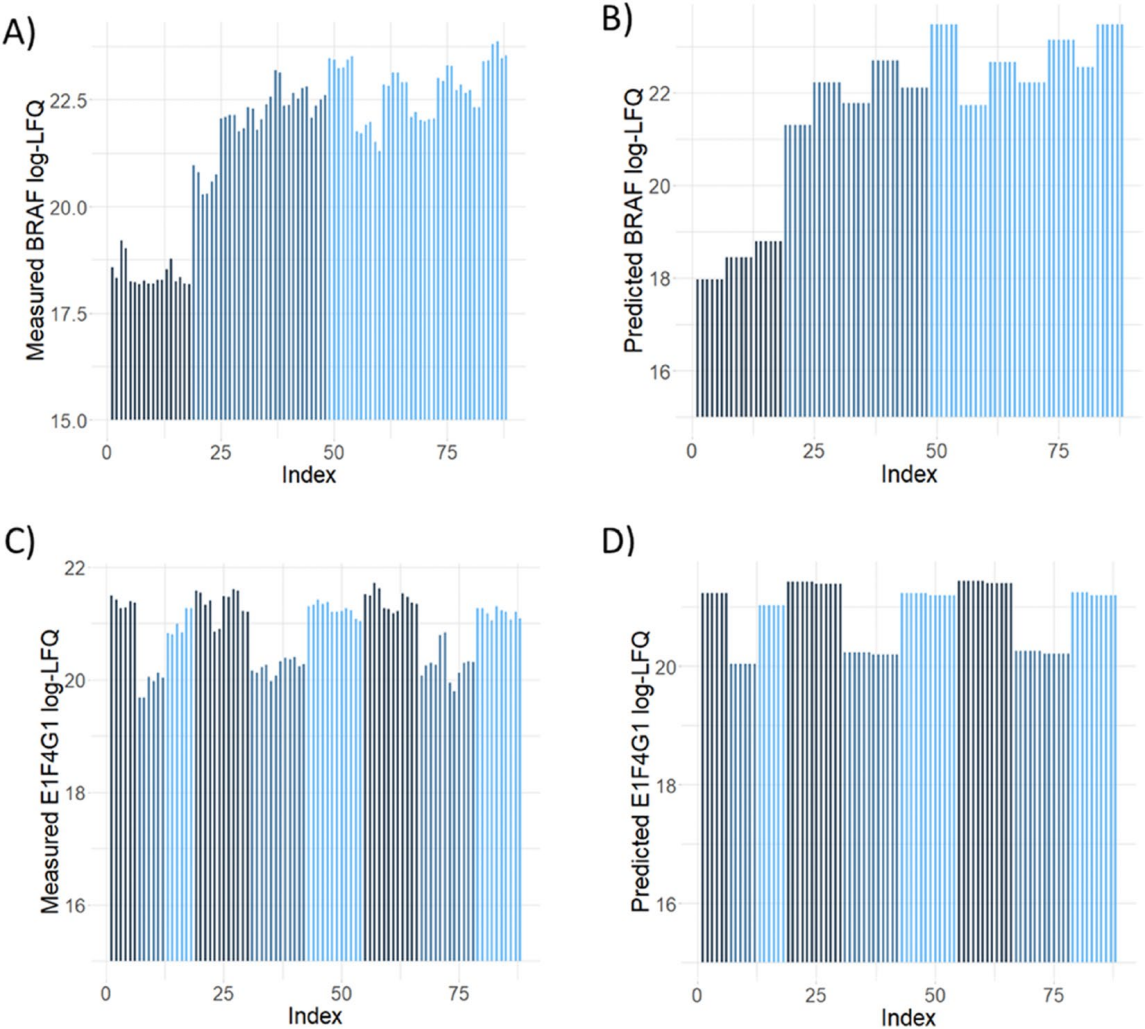
Accuracy of linear regression analysis. Panels (**A**,**B**) show the measured and predicted log-LFQ intensities for BRAF. Black lines represent control transfections, light blue lines samples transfected with BRAFV600E, and grey lines samples transfected with BRAF, respectively. Similarly, panels (**C**,**D**) show the measured and predicted log-LFQ intensities for E1F4G1. Grey lines represent treatment with Sorafenib, blue lines treatment with Vemurafenib, and black lines treatment with DMSO as control.

In order to compare all conditions against each other we used a linear regression analysis to model how treatment, BRAF transfection, and presence of the A/C heterodimerizer affected PPIs. Using a one-hot encoding for each of these conditions we built a linear regression model for each of the proteins found to be significantly overexpressed in at least one condition. We reliably could predict log-LFQ intensity based on the sample conditions, and two examples are shown in Fig. 5. This analysis supports the validity of our regression-based strategy to identify differential PPIs in the dataset.

To compare our dataset with published data, we checked for the overlap between our dataset and the IntAct database restricting the search to protein interactions found in HEK293 cells. Under these conditions the overlap (i) between the IntAct RAF1 and our RAF1 data set is 11 proteins (STUB1, HSPA8, BAG2, CDC37, EIF3D, DYNLL1, HSP90AA1, IRS4, YWHAG, YWHAQ, YWHAH); and (ii) between the IntAct BRAF and our BRAF data set is 7 proteins (YWHAQ, YWHAZ, YWHAE, YWHAG, YWHAH, BRAF, RAF1). As RAF1 was the primary pulldown bait and BRAF was recruited to RAF1 via the dimerizer drug, a smaller number of overlapping BRAF interactors is not surprising. In summary, these results suggest that - while we find known interactors – our dataset adds many previously unknown interactions.

## Data Availability

The functions used to analyse the dataset were deposited in the following repository https://github.com/Luisiglm/proteomics_R.
